# Mortality and Health Outcomes of HIV-Exposed and Unexposed Children in a PMTCT Cohort in Malawi

**DOI:** 10.1371/journal.pone.0047337

**Published:** 2012-10-17

**Authors:** Megan Landes, Monique van Lettow, Adrienne K. Chan, Isabell Mayuni, Erik J. Schouten, Richard A. Bedell

**Affiliations:** 1 Dignitas International, Zomba, Malawi; 2 Department of Family and Community Medicine, University of Toronto, Toronto, Canada; 3 Dalla Lana School of Public Health, University of Toronto, Toronto, Canada; 4 Management Sciences for Health, Lilongwe, Malawi; Africa Centre for Health and Population Studies - University of KwaZulu-Natal, South Africa

## Abstract

**Background:**

Mortality and morbidity among HIV-exposed children are thought to be high in Malawi. We sought to determine mortality and health outcomes of HIV-exposed and unexposed infants within a PMTCT program.

**Method:**

Data were collected as part of a retrospective cohort study in Zomba District, Malawi. HIV-infected mothers were identified via antenatal, delivery and postpartum records with a delivery date 18–20 months prior; the next registered HIV-uninfected mother was identified as a control. By interview and health record review, data on socio-demographic characteristics, service uptake, and health outcomes were collected. HIV-testing was offered to all exposed children.

**Results:**

173 HIV-infected and 214 uninfected mothers were included. 4 stillbirths (1.0%) occurred; among the 383 livebirths, 41 (10.7%) children died by 20 months (32 (18.7%) HIV-exposed and 9 unexposed children (4.3%; p<0.0001)). Risk factors for child death included: HIV-exposure [adjOR2.9(95%CI 1.1–7.2)], low birthweight [adjOR2.5(1.0–6.3)], previous child death (adjOR25.1(6.5–97.5)] and maternal death [adjOR5.3(11.4–20.5)]. At 20 months, HIV-infected children had significantly poorer health outcomes than HIV-unexposed children and HIV-exposed but uninfected children (HIV-EU), including: hospital admissions, delayed development, undernutrition and restrictions in function (Lansky scale); no significant differences were seen between HIV-EU and HIV-unexposed children. Overall, no difference was seen at 20 months among HIV-infected, HIV-EU and HIV-unexposed groups in Z-scores (%<−2.0) for weight, height and BMI. Risk factors for poor functional health status at 20 months included: HIV-infection [adjOR8.9(2.4–32.6)], maternal illness [adjOR2.8(1.5–5.0)] and low birthweight [adjOR2.0(1.0–4.1)].

**Conclusion:**

Child mortality remains high within this context and could be reduced through more effective PMTCT including prioritizing the treatment of maternal HIV infection to address the effect of maternal health and survival on infant health and survival. HIV-infected children demonstrated developmental delays, functional health and nutritional deficits that underscore the need for increased uptake of early infant diagnosis and institution of ART for all infected infants.

## Introduction/Background

In 2010, UNAIDS estimated 20% of all children born in sub-Saharan Africa were exposed to HIV, among whom 130 000 new HIV-infections occurred (90 000–160 000) [1]. Morbidity and mortality burden among these newly HIV-infected infants is high, with an estimated 50% mortality rate by their second year [2]. Of the remaining children who are HIV-exposed but who remain uninfected (HIV-EU), several studies describe elevated mortality in comparison to HIV-unexposed infants [3]–[6]. The mechanism of increased risk among HIV-EU infants and children is not entirely clear, but may be linked to the effects of maternal HIV-related illness and mortality [7], [8], [9], exposure to opportunistic infections [10] and/or a mother/families' capacity to care for infants, such as ability to breastfeed [11].

As part of a retrospective cohort evaluating the uptake and outcomes of prevention-of-mother-to-child-transmission (PMTCT) services in a rural district of Malawi under operational conditions, additional data was collected on child deaths and health outcomes at 18–20 months post-delivery [7]. In Malawi, where PMTCT services began in 2003, approximately 470 000 women older than 15 years are living with HIV [1]. At the time of study (ie. 2008–2009), PMTCT services included routine HIV testing and counseling (HTC) at antenatal clinics (ANC); WHO clinical staging and CD4 count (if WHO Stage I or II and available), initiation of highly active antiretroviral treatment (HAART) for women in WHO clinical stages III or IV, or in stages I and II if CD4 count <250 (up to 2009); single dose nevirapine (sd-NVP) for women not initiated on HAART and for all HIV-exposed infants, and follow-up HTC of exposed infants up to 18 months [10]. We reported previously [7], the suboptimal use of sd-NVP prophylaxis with only 75% of mothers not on HAART taking sd-NVP at onset or during labor and 66% of infants receiving sd-NVP within 72 hours after birth. Additionally, infant follow-up for diagnosis was low as only 28% of HIV-exposed infants ever attended [7].

Given existing literature which points to an association between HIV-exposure and health outcomes in children, we aim to describe the child mortality within this PMTCT cohort and the health outcomes of HIV-infected, HIV-exposed but uninfected (HIV-EU), and HIV-unexposed children at 20 months under routine, operational conditions in a rural district of Malawi.

## Methods

### Study setting and data collection

Data were collected as part of a retrospective cohort study in Zomba District (population 670,000), a primarily rural district in south-eastern Malawi. Antenatal care coverage in Malawi is high (92%) [12]. The methods used in this study have been described in detail elsewhere [7]. HIV-infected mothers who attended one of the 20 rural public facilities that provide antenatal services, and had an estimated or documented delivery date (EDD) between March 1st and May 31st 2008, were identified for inclusion through antenatal registers. Additionally we reviewed all delivery and postpartum registers during the specified period in an attempt to capture all HIV-infected pregnant women in the district with an EDD between March 1^st^ and May 31^st^, 2008 . For every HIV-infected mother, the next registered HIV-uninfected mother was identified as a control.

Community health workers, known as Health Surveillance Assistants (HSAs), already working for the Ministry of Health in the catchment areas of the individual rural health facilities helped trace the identified women for the purpose of this study, and then asked the identified mother-child pairs to come to the health centre for interviews. In cases where the mother had died, the child's primary caregiver was invited to participate. In cases where identified women were reported to have moved or died, village headmen were consulted for confirmation and village registers reviewed for verification.

Data collection was conducted by 6 trained female interviewers, through semi-structured interviews using a standardized questionnaire. Information concerning prior HIV-testing and ART was verified through personal health passports.

Specifically for this analysis, socio-demographic information was collected, including socio-economic indicators (i.e. level of education, housing type, water source, household size, source of income and means of transport). Socio-economic indicators were selected using the household poverty assessment model for Malawi as described by Payongayong et al. [11] The selected variables were ranked from poorest to wealthiest; a rank sum of all the ranked variables was made and categorized in percentiles, with 1 as the poorest and 4 the wealthiest. Data on health outcomes was collected on mothers and children surviving to 18–20 months (ie. the time of study); there was no information available on health status for those mothers or children who had died prior to the time of study. Women were asked in their mother tongue (Chichewa) to report their own and their child's perceived health on a comparative qualitative scale as “excellent”, “good”, “fair” or “poor”. Interviewers then ranked the functional health status of the child on the Lansky scale based on a series of questions which describe the activity restrictions of the child secondary to disease symptoms [13]. Age-appropriate developmental benchmarks were identified by a pediatrician, with the advantage of being simply understood and dichotomous in their answers. We used as benchmarks of development a child of 18–20 months who (i) cannot walk or (ii) cannot fetch his dish when asked to as demonstrating (i) gross motor delay or (ii) cognitive delay. Weight, height and mid-upper-arm circumference (MUAC) were obtained at the time of interview.

HIV-infection status of children was ascertained from health passports at the time of study. All mothers and/or children with negative or unknown HIV status were offered point of care HIV rapid testing and those testing positive were referred for assessment of ART eligibility.

### Statistical analysis

Data and statistical analysis were conducted using STATA 11.0 (StataCorp, Texas, USA). Characteristics were summarized using means or medians for continuous variables and proportions for categorical variables and compared using Student t-tests, non-parametric tests and Chi-square tests, respectively. Z-scores for weight, height and BMI were calculated to examine growth among unexposed, exposed uninfected and exposed infected children. Weights and heights of our cohort of unexposed/uninfected children were used as reference, and comparisons were made between proportions of z-scores <−2.00. [14] Confidence intervals were derived for comparison of health outcomes amongst HIV-unexposed, HIV-exposed/uninfected (HIV-EU) and HIV-infected infants. Relative risks (RR) were used as a measure of associations between HIV status and functional health status. Risk factors for reporting of functional health restrictions were determined through multivariable logistic regression, described as adjusted odds ratios (aOR).

### Ethics Approval

This study received ethical approval from the National Health Science Research Committee in Malawi. Written informed consent was given by all participants.

## Results

### Characteristics of HIV-infected and uninfected mothers and children

360 HIV-infected mothers and 360 HIV-uninfected mothers were identified through all available registers. 173 HIV-infected and 214 HIV-uninfected mothers were found and included in the study. Out of the 720 mother-child pairs traced, 61 had moved out of the area, 269 unable to be found and 3 were not willing to participate. No significant difference was found between HIV-infected and HIV-uninfected women in the proportion having moved out of the area or not found (p = 0.09).

Baseline characteristics of the 173 HIV-infected and 214 uninfected mothers are reported previously from this study [7]. HIV-infected mothers were older [29.1 years (±SD 5.8 years) vs. 25.3 years (±SD 5.6); p<0.001] and of higher parity [4.5 (±SD 2.1) vs. 3.1(±SD 2.0); p<0.001] than HIV-uninfected women. The association between parity and age stratum was similar for HIV-infected and –uninfected women. No significant differences were found in terms of employment status, dwelling type, water source or transport method used, indicating a uniformly poor community [7].

We reported previously that among those HIV-infected women, only 10% of HIV-infected mothers were on HAART before delivery and 27% by 18–20 months post-partum. sd-NVP was taken by 75% of HIV-infected mothers not on HAART, and given to 66% of infants. 18% of HIV-infected mothers followed all current recommended PMTCT options.

Additionally, we previously reported that only 28% of HIV-exposed children were documented to have followed up for early infant diagnosis (EID). [7] Of those surviving to 18–20 months and included in this cohort, 7 children were confirmed to be HIV-infected and 2 were currently on ART. At the time of study, testing was offered to HIV-exposed children with unknown or previously negative status and eight new infections were identified, giving a total number of transmissions among living tested children as 15/111 (13.5%).

### Breastfeeding practices

We previously described [7] that HIV-infected women were more likely to opt for 6 months exclusive breastfeeding (as recommended at time of study) compared to HIV-uninfected women; 20% versus 1% respectively (p = 0.002). The majority of exposed and unexposed children received mixed feeding from 3 months of age. Unexposed children were breastfed longer than exposed children (mean 18 vs. 12 months, respectively; p<0.01), and 48% of exposed and 90% of unexposed children were still breastfed at >18months (p<0.01).

### Child Mortality

Among the 387 mother-child dyads identified, there were 4 stillbirths (1.0%); 2(1.2%) in HIV-infected women and 2 (0.9%) in HIV-uninfected women (p = 0.83). Among the remaining 383 livebirths, there were 41 (10.7%) child deaths by 20 months; 32 (18.7%) occurring in HIV-exposed children and 9 (4.3%) in HIV-unexposed (p<0.0001; see Table 1). Note that we were unable to distinguish between HIV infected and HIV-exposed but uninfected children who had died since none were tested for HIV infection. Mortality risk among HIV-exposed infants (by 12 months) was estimated at 129 deaths/1000 livebirths and by 20 months at 187/1000 livebirths.

**Table 1:** Child Mortality amongst HIV-infected and HIV-uninfected mothers.

		HIV-infected mothers N = 173	HIV-uninfected mothers N = 214	P-value
**Infant Death (12 mo)** * **n (%)**		22 (12.8)	7 (3.3)	<0.001
**Child Death (20 mo)** * **n (%)**		32 (18.7)	9 (4.3)	<0.001
**Age at child death** * **median months (IQR)**		9.2 (2.5–14.6)	5.9 (4.4–7.0)	0.05
**Interval Mortality Rates** * n (%)	0–7 days	5 (2.9)	1 (0.5)	0.83
	7–28 days	3 (1.8)	0	0.09
	1–12 mos.	14 (8.2)	6 (2.8)	0.03
	12–20 mos.	10 (5.9)	2 (0.9)	0.02
**Total Lifetime Risk of Child death (deaths/1000 livebirths)**		195	135	0.005
***By maternal age*** *** ***(years)***	15–20	125	94	0.72
	21–25	224	112	0.02
	26–30	241	93	<0.0001
	31–35	200	211	0.80
	>35	129	196	0.24

* Amongst livebirths (excluding stillbirths).

*** Maternal age at time of study.

**Figure 1:**
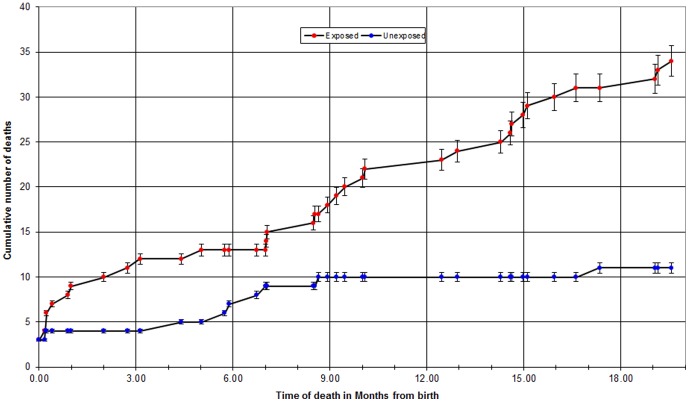
Cumulative mortality among HIV-exposed children versus HIV-unexposed children over time.

The median age at infant death for HIV-exposed children was 9.2 months (IQR 2.5–14.6) versus 5.9 months (IQR 4.4–7.0) for HIV-unexposed children (p = 0.046). In *Figure 1* we show the increased cumulative mortality among HIV-exposed children versus HIV-unexposed children over time; increased risk of death among HIV-exposed children was significantly demonstrated for those greater than one month of age.

We previously reported 4 seroconversions in this cohort of women who were documented HIV-uninfected at the time of ANC registry or labor ward and were tested positive by the study team at 18–20 months; the time of conversion was unknown. Among the four infants born to these women, one died at the age of 6 months. The HIV-free survival rate in this cohort was estimated at 66% (95%CI 58–74) [7].

### Overall burden of child mortality

We collected data on the total number of child deaths all women had experienced; there were significant differences between HIV-infected and HIV-uninfected women in having had previous child deaths prior to this index pregnancy. When controlling for age and parity, HIV-infected women were more likely to have had a previous child die than HIV-uninfected mothers at the following intervals: less than 48 hours [adjOR 2.2 (95%CI 1.0–4.7); p = 0.046] and less than 12 months [adjOR 2.3(95%CI 1.3–4.0); p = 0.004], but not between 1 and 5 years old (p = 0.312).

In addition, we collected data from all mothers on the total number of previous child deaths they each had (0–5 years of age), along with the total number of livebirths; age-stratified data is reported in *Table 1*. The overall lifetime risk of child death among all livebirths was significantly higher among HIV-infected women (p = 0.005), however in the age-stratified data we report that there was no difference among younger women (15–19) and older women (31–45); limitations in the data do not allow us to control for timing of HIV-infection and/or parity, although there was no significant difference in parity among those HIV-infected and HIV-uninfected women aged 30–34 (p = 0.035)and >35 years (p = 0.76).

### Risk Factors for Child Death

In *Table 2* we report the multivariable analysis for risk factors associated with a death in the index child during this study. HIV-exposure tripled the risk of child death (p = 0.03), low birthweight more than doubled the risk (p = 0.05) and a previous child death increased the risk of death in this index pregnancy by 25-times (p<0.0001). Maternal death was significantly associated with a five-fold increase in the risk of child death when controlling for HIV-exposure and maternal age. Marital status, socio-economic status, BMI, and maternal self-reported health status (at the time of the study) were not associated with child death.

**Table 2:** Risk Factors for Child Death.

		Unadjusted OR (95%CI)	Adjusted OR (95%CI)	p-value
**HIV-exposure** 1 ^,^ 2	Unexposed	1.0	1	
	Exposed	5.2 (2.4–11.2)	2.9 (1.1–7.2)	0.03
**Previous Child Death (<5 yrs)** 3	None	1	1	
	At least one	15.3 (5.3–44.5)	25.1 (6.5–97.5)	<0.001
**Birthweight** 4	>2500 g	1.0	1	
	<2500 g	2.9 (1.3–6.8)	2.5 (1.0–6.3)	0.05
**Maternal Death** 5		7.8 (2.3–26.8)	5.3 (1.4–20.5)	0.02

1 HIV-exposed is considered those children with known exposure from birth (ie. mother was tested in ANC or on the labour ward).

2 Controlled for previous child death, birth weight, age.

3 Controlled for HIV-exposure, birthweight, age.

4 Controlled for HIV-exposure, previous child death, age.

5 Controlled for HIV-exposure, age (information not available for birthweight and previous child death for those mothers who had died).

When limiting the analysis to the 171 livebirths of HIV-infected mothers (status known at birth), there was no further association between child death and maternal uptake of PMTCT services (i.e. HIV testing, maternal sd-NVP receipt/use, infant sd-NVP use and exclusive breastfeeding; all p>0.05) nor current (i.e., at 20 months post-partum) maternal ART use (p = 0.06). Child ART use was not included in the model, as only 2 of the HIV-infected children had started ART. Information regarding WHO staging of mothers or CD4 count was not available in this study.

### Health Outcomes Among HIV-infected, HIV-exposed but uninfected, and HIV-unexposed children surviving to 18–20 months

In *Table 3*, we report both objective measures of health status as well as maternal reporting of child health status (both perceived health status and functional health status on the Lansky scale) among those HIV-infected, HIV-exposed but uninfected (HIV-EU) and HIV-unexposed children surviving to 20 months.

**Table 3:** Health outcomes of children surviving to 20 months by HIV-infection status.

	HIV-unexposed (N = 200) (%)(95%CI)	HIV-exposed, uninfected (N = 128)^1^ (%)(95%CI)	HIV-infected (N = 14)(%)(95%CI)
**Birthweight**			
Low <2500 g	15 (10–20)	18 (11–25)	33 (2–65)
Normal >2500 g	85 (80–90)	82 (75–90)	67 (35–98)
**Episode in the past 3 months of:**			
Diarrhea	57 (50–64)	49 (40–58)	77 (50–100)
Cough	55 (48–62)	62 (53–70)	23 (0–50)
Skin Rash	19 (14–25)	16 (10–23)	23 (0–50)
Oral Thrush	25 (19–31)	19 (12–26)	8 (0–24)
Fever	82 (77–88)	77 (70–85)	85 (62–100)
**Times admitted to hospital ever**			
0	79 73–85)	74 (66–81)	71 (44–99)
1–3 times	20 (14–25)	26 (19–34)	14 (0–35)
≥3 times	2 (0–3)	.	15 (0–35)
**Development Indicators**			
Walking	96 (92–98)	91 (86–96)	64 (36–93)
Understands	94 (91–97)	94 (91–98)	64 (35–93)
**Z-scores for weight (% <−2.0)**	1.0 (0–2.4)	4.0 (0–7.4)	7.4 (0–22.6)
**Z-scores for height (%<−2.0)**	2.5 (0–4.7)	4.0 (0–7.4)	7.4 (0–22.6)
**Z-scores for BMI (%<−2.0)**	1.0 (0–2.4)	4.0 (0.7.4)	5/5 (100%)
**MUAC**			
Green	97 (95–99)	93 (88–98)	86 (65–100)
Yellow/Red	3 (0–5)	7 (3–12)	14 (0–35)
**Child Health Status (per maternal report)**			
Excellent	49 (42–56)	39 (31–48)	29 (2–56)
Good	40 (33–47)	38 (29–46)	43 (13–73)
Fair	11 (6–15)	22 (15–29)	21 (0–46)
Poor	-	2 (0–4)	7 (0–23)

1 Including 3 children born to women with seroconversion identified in the study.

Overall, HIV-EU children showed no significant increased risk of worse outcomes amongst our measures, however HIV-infected children showed significantly worse objective measures of health at 20 months than HIV-EU or HIV-uninfected children,

Among development indicators, HIV-infected children were more likely to have some gross motor delay (ie. not walking; 64% vs 91% and 96%; both p<0.001) and gross cognitive delay (ie. not understanding simple, age-appropriate commands (64% vs. 94% for both; both p<0.001)) than HIV-EU and/or HIV-unexposed children. There were no significant differences found between HIV-EU children vs. HIV-unexposed children in walking (p = 0.13) or understanding (p = 0.93).

We measured MUAC as an estimate of undernutrition; HIV-infected children were almost six times more likely to have a low MUAC (yellow/red) than HIV-unexposed children [RR 5.7 (95% CI 1.2–26.7); p = 0.02]. There was a borderline significant increased risk of a low MUAC score among HIV-EU versus HIV-unexposed children (7% vs 3%; p = 0.06). There were no significant differences seen in the percent of children with low z-scores for weight, height and BMI between all HIV-status categories at 20 months.

When mothers were asked to rank their child's health, 152 mothers ranked their children's health as “excellent”, 134 as “good”, 53 as “fair” and 3 as “poor”. Maternal reporting of worse child health (ie. less than “excellent”) was associated with objective measures of poor child health: low BMI (p = 0.02), a yellow or red MUAC (p = 0.02 and p = 0.003), failure to reach development milestones [i.e. not walking (p = 0.003) and not understanding commands (p = 0.003)], and times admitted to hospital (p<0.0001). Additionally, mothers' ranking of child health was well correlated with the child's functional health status on the Lansky scale (p<0.0001).

On the functional health scale, HIV-infected children were eight times more likely to have minor restrictions in physical activity [OR 8.1 (95%CI 2.0–32.0);p = 0.003] and over 20 times more likely to have moderate [OR 28 (95%CI 3.6–216);p = 0.001] or major restrictions of activity [OR 21 (95%CI 1.5–281.9);0.02] than HIV-unexposed children. There was no significant difference in functional health status between HIV-EU children and HIV-unexposed children (p = 0.27).

In *Table 4* we report risk factors for poor functional health status. HIV-infected children were almost nine-times more likely to have any restrictions in functional health (p = 0.001), while HIV-EU children had no significant increased risk. Maternal illness (p = 0.001) and low birthweight (p = 0.05) were also significantly associated with functional health restrictions. In addition, among the subset of HIV-exposed children, maternal uptake of PMTCT services was not associated with functional health status of children surviving to 20 months.

**Table 4:** Risk Factors for Functional Health Restrictions Among Children Surviving to 20 months.(1)

		Normal/Active Child n (%)	Minor or major restrictions in child's activity n (%)	Unadjusted OR (95%CI)	Adjusted OR (95%CI)
**HIV status** a	Unexposed	168 (62.7)	31 (42.5)	1	1
	Exposed/uninfected	96 (35.8)	32 (43.8)	1.8 (1.0–3.1)	1.4 (0.7–2.5)
	Infected	4 (1.5)	10 (13.7)	13.5 (4.0–45.9)	8.9 (2.4–32.6)
**Maternal Health** a	Alive and healthy	204 (76.1)	35 (48.0)	1	1
	Alive with minor or major symptoms of disease	61 (22.8)	3 (4.1)	3.3 (1.9–5.8)	2.8 (1.5–5.0)
	Mother Dead	3 (1.1)	35 (48.0)	5.8 (1.1–30.0)	2.9 (0.4–19)
**Birthweight** a	Normal	211 (86.1)	48 (72.7)	1.0	1
	Low	34 (13.9)	18 (27.3)	2.3 (1.2–4.5)	2.0 (1.0–4.1)

(1) Based on Lansky Functional Health Scale.

a Controlled for all other variables in the table.

## Discussion

Our results indicate high mortality at 20 months among HIV-exposed infants (187 deaths/1000 livebirths), consistent with a meta-analysis in sub-Saharan Africa (SSA) reporting a cumulative mortality rate of 174/1000 livebirths among HIV-exposed children at 24 months [15].

The unique value of this study is its comparison to a reference population of HIV-unexposed children in Zomba District; we show a greatly increased risk of mortality at all key intervals from 1 month to 20 months for HIV-exposed children. Overall, by 20 months, mortality was more than four times greater among HIV-exposed than among HIV-unexposed children. This increased risk of mortality for HIV-exposed children is similar to other cohorts within SSA where estimates have ranged from 2 to 8-fold increased risk of death by 24 months for HIV-exposed infants [3]–[6].

We previously report a low uptake of early infant diagnosis in this study (28%) which, in this operational setting, translates into an inability to comment on which of the infants were HIV-infected that died [7]. We are thus unable to determine whether HIV exposure (without infection) could also be associated with risk of child death and it remains possible that our reported mortality risk is secondary to HIV-infection or simply HIV-exposure. While HIV-infection itself is well documented to dramatically increase the risk of death for children under two years [2], [15], several prospective studies have also demonstrated an independent increased risk of mortality for HIV-exposed but uninfected children by two years of age [16]–[18]. HIV-exposure alone may increase mortality risk via unmeasured impacts of HIV-related illness on the family (i.e. socioeconomic or care giving), exposure to maternal opportunistic infections (i.e. tuberculosis) or deleterious effects on breastfeeding [19], [20]. We highlight this importance of this discussion as 12 million HIV-infected women live in SSA, many of whom are multiparous, and thus excess child mortality attributed to HIV-exposure alone, regardless of infant HIV-infection status, stands to have a significant population level impact [1]. Special attention is deserved towards improving a comprehensive PMTCT cascade which ultimately includes the prevention of infection in potential parents, unwanted pregnancies and proven interventions to reduce the transmission during pregnancy, childbirth and breastfeeding.

When comparing health outcomes among HIV-infected, HIV-exposed but uninfected (HIV-EU) and HIV-unexposed children who survived to 20 months, we report an increased risk of poor health outcome measures among surviving HIV-infected children. Across the range of outcomes measured, this is to be expected given supporting studies in the literature: a recent systematic review showed HIV-infected children to be noticeably delayed in early motor and language development by the age of two years [21] , multiple studies show increased risk of opportunistic infections [22], [23] and admission to hospital [19] and several demonstrate risk of undernutrition [24], [25]. It should also be noted that our HIV-infected cohort was predominantly not on ART, highlighting the low uptake of ART among those with early infant diagnosis and the missed opportunity for ART among the new infections identified in the study at 18–20 months. The poor health outcomes we report for HIV-infected children in this setting along with these missed opportunities for ART application supports the strengthening of early infant diagnosis and ART referral.

Additionally, we do not report any significant increased risk of poor health outcome measures for HIV-exposed but uninfected children surviving to 18–20 months. In our setting, where there is an antenatal HIV prevalence of approximately 12–24%, the HIV-exposed but uninfected constitutes a generous proportion of the pediatric population [26]. Given the retrospective data collection we are unable to assess the health outcomes of those HIV-exposed children who had died prior to the study, however our lack of significance in health outcomes may imply the greatest risk of HIV-exposure on mortality and morbidity may lie before 18–20 months of age.

A strong link between maternal health and survival and child health and survival was demonstrated in this study. This adds support to the existing literature [2], [3], [4], [6], [8], [9] and highlights the importance of targeting improvements in maternal health within maternal and child health (MCH) *and* PMTCT policy and programming for both HIV-infected and HIV-uninfected mothers. We previously report in this cohort suboptimal uptake of ART among HIV-infected mothers [7] and targeted programmatic improvement within our context should include improved early detection, referral and treatment with ART. As Malawi moves towards a universal testing and lifelong treatment strategy for all pregnant women (know as Option B Plus) this should begin to improve ART coverage for all women with HIV-infection [27].

Strikingly, HIV-infected women had much higher mortality among their previous children than HIV-uninfected women, indicating the large lifetime burden of child mortality that may be associated with HIV-infection. In a cohort of HIV-infected pregnant women in Zambia, a previous infant death was associated with an increased risk of low CD4 count (<350 cells/mm3) [28] and thus may be a marker of advanced HIV disease and confer the associated risk of death through this mechanism, or may increase the risk of death through some other unmeasured risk factor. In addition, having had at least one previous child death remained a risk factor for child death in this index pregnancy, regardless of maternal HIV status. A similar finding was shown in a study examining mortality and birth spacing among HIV-uninfected women in Malawi, where there was an increased risk of infant death if the preceding child had also died [29]. Regardless of HIV status, screening for previous infant death by health care providers may provide a useful mechanism to identify high risk mother-child dyads and appropriately allocate resources.

## Limitations

This study was limited to the cohort of women traced, with significant loss to follow-up of both HIV-infected and uninfected women. While these proportions were not significantly different, we cannot comment on the characteristics of those women and children loss to follow-up. This may have lead to an underestimation of mortality as several studies have shown cohorts of loss-to-follow-up patients can have significant rates of both. Additionally, this loss to follow-up will lead us to a selection bias in reporting of health outcomes and we were only able to describe the health status of those children surviving to 18–20 months. This selection bias may be the reason for lack of associations seen in health measures (ie. such as z-scores) amongst HIV-infected, HIV-EU and HIV-unexposed infants as these represent surviving children and not the entire cohort from delivery. Another possible reason for lack of associations may be explained by limited statistical power due to small number of events in some categories (ie. such as child death and maternal ART).

Given the retrospective data collection, maternal recall may bias the data collected regarding subjective health outcomes. However, we attempted to validate these measures by correlating subjective measures (ie. perceived health status) with objective measures (ie. MUAC or Lansky functional scale). Additionally, we were unable to conduct a full developmental assessment of these children, and we recognize that the reported measures are at best a benchmark in assessment.

Finally, we have discussed above the low uptake of early infant diagnosis and also report few measures of CD4 counts and viral loads for both mothers and children in this study, which is a reflection of the operational setting in Malawi. We continue to assert that lack of proper staging and CD4 count ascertainment in this operational setting resulted in under identification of more advanced HIV-infection and underapplication of ART.

## Conclusions

Infant & child mortality could be dramatically reduced through effective PMTCT coupled with treatment of maternal HIV infection. This addresses not only vertical transmission of HIV but the effect of maternal health (and survival) on infant health and survival.

A simple antenatal screening question regarding prior infant or child deaths quickly identifies women with increased risk of subsequent infant or child death and could prompt more careful follow-up of high-risk women and infants. HIV-infected children demonstrate developmental delays, functional health and nutritional deficits that underscore the need for improved early infant diagnosis of HIV and immediate institution of ART for all infected infants.
